# Supplementary material for: Rhythmic properties of Sciaena umbra calls across space and time in the Mediterranean Sea
Source: PLoS One. 2024 Feb 21;19(2):e0295589. doi: 10.1371/journal.pone.0295589 (PMC10881014; doi:10.1371/journal.pone.0295589)
Supplement: S1 File — (DOCX) [file pone.0295589.s001.docx]

**Supplementary Materials: Rhythmic properties of *Sciaena umbra* calls across space and time in the Mediterranean Sea**

Marta Picciulin^1^, Marta Bolgan^2^, Lara S. Burchardt ^*3,4^

^1^CNR-National Research Council, ISMAR - Institute of Marine Sciences, Venice, Italy

^2^Ocean Science Consulting Limited, Dunbar, United Kingdom

^3^Max-Planck-Institut for Psycholinguistics, Nijmegen, Netherlands

^4^Leibniz-Zentrum Allgemeine Sprachwissenschaft, Berlin, Germany

*Corresponding author; e-mail: [l.s.burchardt@gmx.de](about:blank)

1. Theoretical Considerations

2. Generalized Additive Models (GAMs)

1. Theoretical Considerations

Table S1: Theoretical Considerations on which variables might influence sound rhythms in fish

| Variable | Hypothesis | Prediction | Variable in dataset |
| --- | --- | --- | --- |
| Temperature (T) | Temperature affects the contraction speed dynamics of sonic muscles. | increasing T 🡪 increasing beat | Temperature in °C per sequence, different precision and resolution between locations. Also, T, was measured at different depths between locations. |
| Time of the day | Diel cycle of sonic activities influences rhythmical property | Sounds emitted during the time with the highest call rate 🡪  higher beat | Categorical, hourly value |
| Boat traffic | The presence of noise from boat traffic affects rhythmical emission | Higher noise level 🡪 higher IOI (vocal compensation) | Value per month/year and location; vessel passes per hour, per m², per month  [h/m²/month]  Several different boat categories:   - Total vessel density - Passenger ships - Recreational boats - Fishing boats - Trade ships - “Other” Vessel types |
| Overall acoustic community diversity | Rhythm patterns are another axis of the realized acoustic niche of a fish species | Higher acoustic richness🡪 lower IOI variability | Acoustic richness, integer,  Number of fish sound types in each location |
| Season | Season affects the rhythmical properties  (i.e. breeding season vs non-breeding season) | Breeding season 🡪 higher beat | Month and day of recording |

1. Generalized Additive Models (GAMs)

The following variables were considered for modelling the IOI beat [Hz]:

- recoding month
- recording day
- acoustic richness
- temperature
- 6 groups of vessel densities in hours/m²/month:
  - total vessel density (vess_den_tot)
  - trading ships (vess_den_trade)
  - fishing ships (vess_den_fishing)
  - passenger ships
  - smaller recreational or pleasure boats
  - “other” boat types

However, as total vessel density highly correlates (r>0.65) with the densities for passenger ships, recreational boats and “other” vessel types, only total vessel density was included in an initial model.

The interaction between day and month was added to the formula.

GAMs were calculated in R using the “mgcv” package, function: gam with the default parameters unless stated otherwise.

**GAMs 1: Full**

ioi_beat ~ s(day) + acoustic_diversity + s(vess_den_trade, k = 9) + s(vess_den_fishing, k = 7) + s(vess_den_tot) + s(temperature) +

s(time_h, k = 4) + month + s(day, by = month)

**GAMs 2: Full – time_h**

ioi_beat ~ s(day) + acoustic_diversity + s(vess_den_trade, k = 9) + s(vess_den_fishing, k = 7) + s(vess_den_tot) + s(temperature) +

month + s(day, by = month)

**GAMs 3: Full – month**

ioi_beat ~ acoustic_diversity + s(vess_den_trade, k = 9) + s(vess_den_fishing, k = 7) + s(vess_den_tot) + s(temperature) +

s(time_h, k = 4) + s(day, by = month)

**GAMs 4: Full – month – time_h**

ioi_beat ~ acoustic_diversity + s(vess_den_trade, k = 9) + s(vess_den_fishing, k = 7) + s(vess_den_tot) + s(temperature) + s(day, by = month)

**GAMs 5: Full – month – time_h – vess_den_tot**

ioi_beat ~ acoustic_diversity + s(vess_den_trade, k = 9) + s(vess_den_fishing, k = 7) + s(temperature) + s(day, by = month)

Table S2: Generalized Additive Models: Five different models. For categorical values, “Estimate” (Est) and p-value (p) are given, while for smoothed continuous variables, F-Statistic and p-values are reported (F/p). Model GAMs 3, highlighted in light green, shows the best combination of indicators and is reported in the main manuscript. GCV: Generalized Cross Validation; AIC: Akaike Information Criterion.

| Model | GCV | AIC | Adjust.  R² | Explained  Deviance | Scale est. | Intercept  (p-value) | month  Est/p | Day  F/p | Interaction day by month  F/p | Time h  F/p | Vess_tot  F/p | Vess_trade  F/p | Vess_fishing  F/p | Acoustic richness  Est/p | Temperature  F/p |
| --- | --- | --- | --- | --- | --- | --- | --- | --- | --- | --- | --- | --- | --- | --- | --- |
| GAMs 1 | 0.004 | -209.66 | 0.67 | 77.2 % | 0.003 | -0.36 (0.04) | 0.1/ 0.07 | 2.33/  0.053 | 2.78/0.04* | 2.08/  0.16 | 3.09/0.09 | 28.91/0.00*** | 14.32/0.00*** | 0.02/0.00*** | 2.03/0.06 |
| GAMs 2 | 0,004 | -208.75 | 0.66 | 76.4 % | 0.003 | -0.39 (0.03*) | 0.1/  0.07 | 2.33/  0.06 | 2.74/0.053 | -- | 2.7/0.1 | 28.41/0.00*** | 14.15/0.00*** | 0.02/0.00*** | 2.12/0.0504 |
| GAMs 3 | 0.004 | -209.66 | 0.67 | 77.2 % | 0.003 | -0.36 (0.04*) | -- | 2.33/  0.053 | 5.12/0.001** | 2.08/  0.16 | 3.09/0.09 | 28.91/0.00*** | 14.32/0.00*** | 0.02/0.00*** | 2.03/0.06 |
| GAMs 4 | 0,004 | -208.75 | 0.66 | 76.4 % | 0.003 | -0.39 (0.03*) | -- | 2.33/  0.06 | 5.49/0.00*** | -- | 2.7/0.1 | 28.41/0.00*** | 14.15/0.00*** | 0.02/0.00*** | 2.12/0.0504 |
| GAMs 5 | 0.004 | -204.88 | 0.64 | 73.4 % | 0.003 | -0.30  (0.089 | -- | 2.49/  0.03* | 5.08/0.001** | -- | -- | 26.25/0.00*** | 13.21/0.00*** | 0.02/0.00*** | 1.52/0.001** |

Models GAMs 1 and GAMs 3 have the same scores, we choose to report on GAMs 3 because it achieves this score with one fewer variable: month. Month as an important season indicator is still present in the model though as the interaction between day and month.


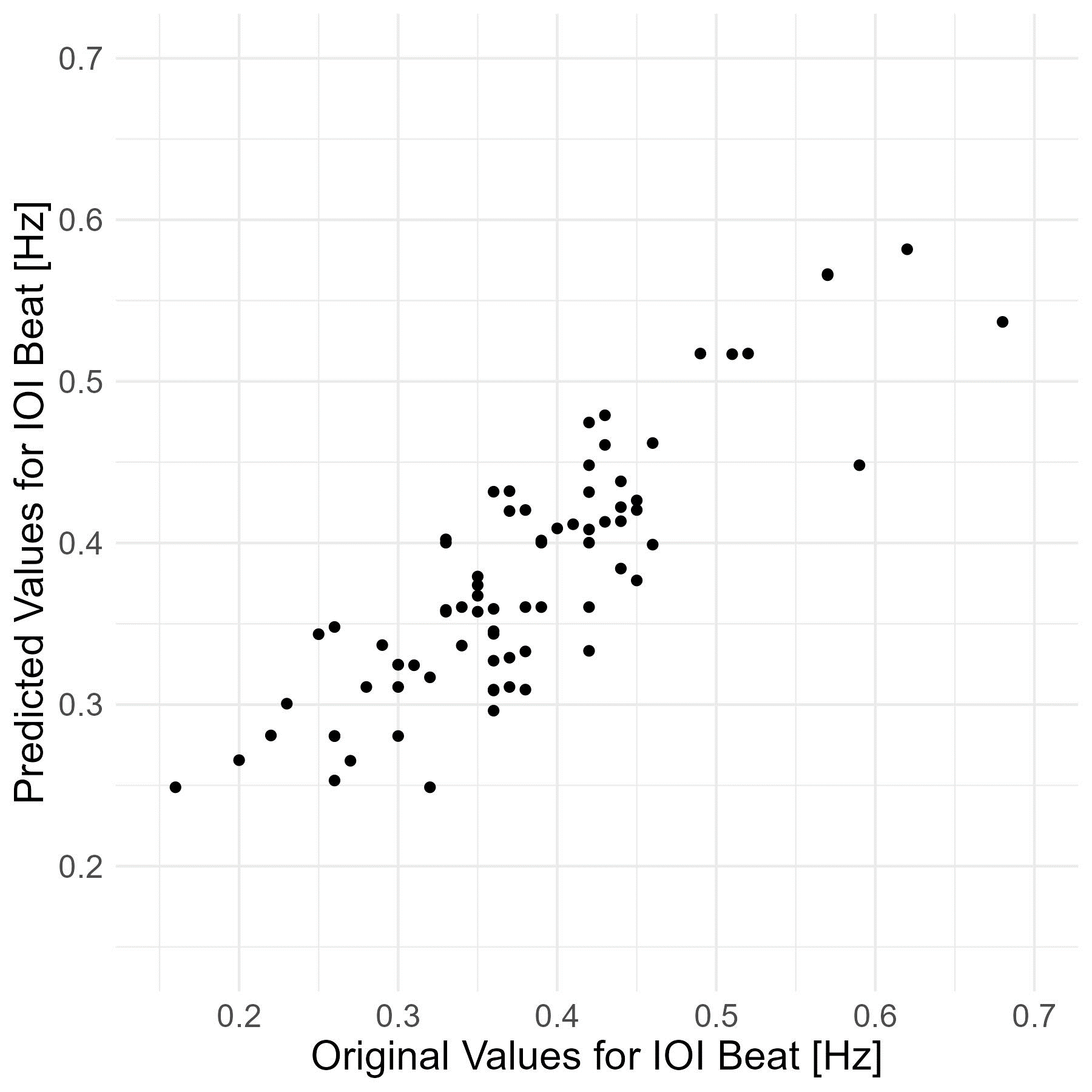


Supplementary Figure 1: Original Values of analyzed IOI Beats [Hz] plotted against the IOI Beat values predicted by the model GAMs 3. The model predicts IOI Beats very well.

In the following IOI beats [Hz] are depicted against the two smoothed parameters showing a significant impact: vessel density of fishing and trading ships. As there is no estimate for the smoothed values, it was checked whether they have a positive or a negative influence on the IOI beat [Hz] visually.


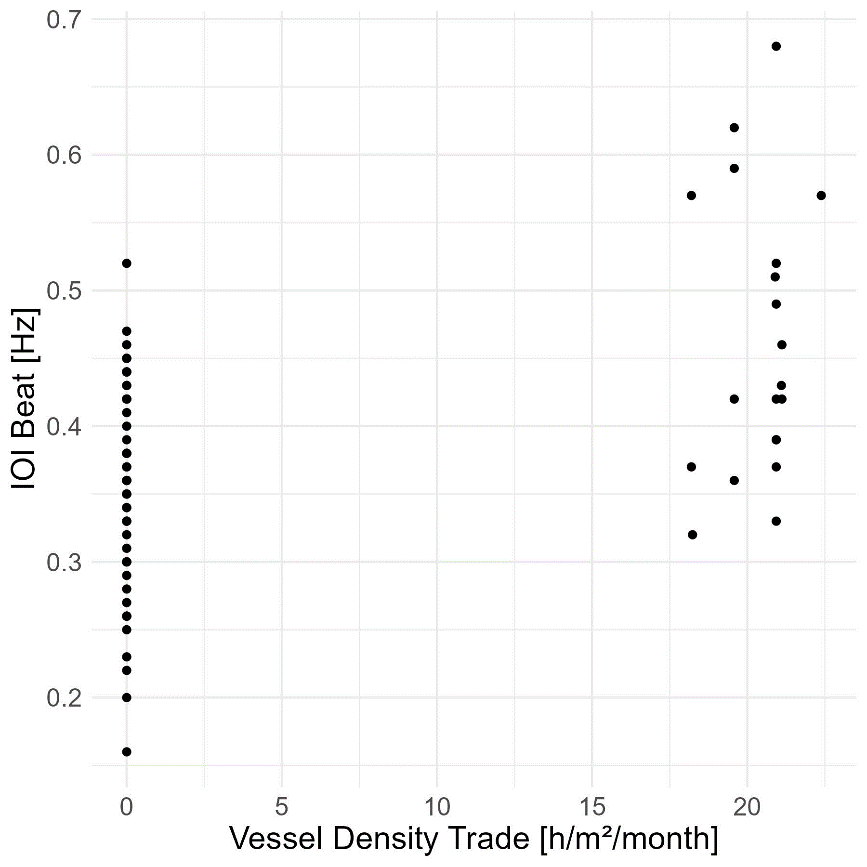

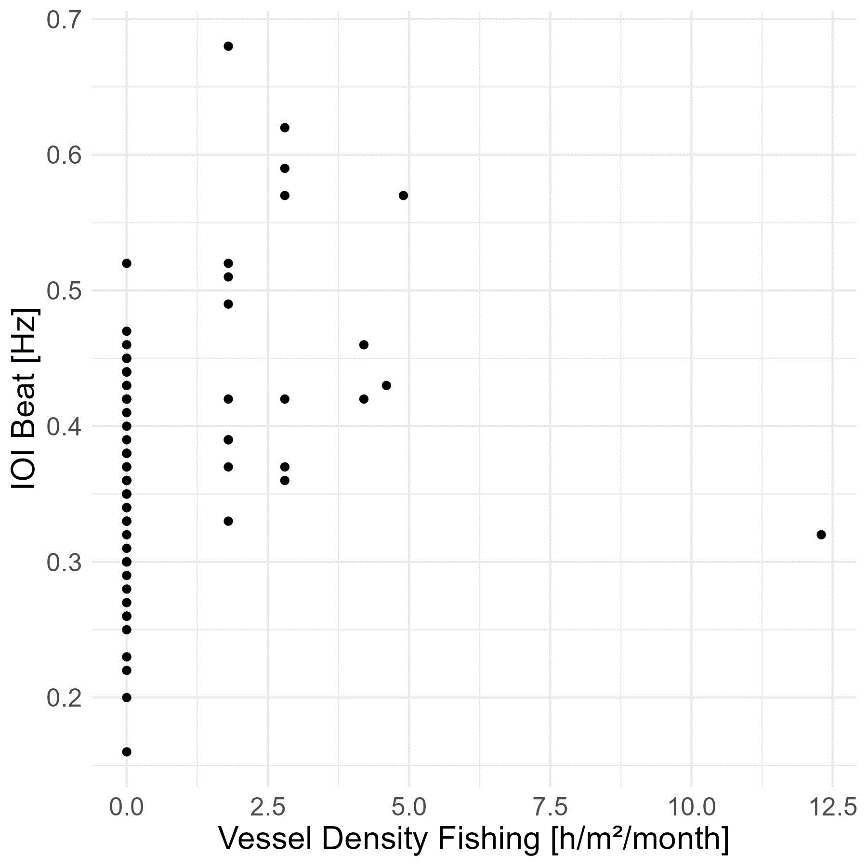
Supplementary Figure 2: IOI Beat in relation to the vessel density of fishing boats. The many 0 values and an outlier (at x > 12) influence the picture, but a general increasing trend can be hypothesized, i.e. higher fishing vessel density leads to higher IOI beats [Hz].

Supplementary Figure 3: IOI Beat in relation to the vessel density of trading ships, i.e. huge cargo ships. The many 0 values influence the picture, but a general increasing trend can be hypothesized, i.e. higher trading vessel density leads to higher IOI beats [Hz].
