# Supplementary material for: Rhythmic properties of Sciaena umbra calls across space and time in the Mediterranean Sea
Source: PLoS One. 2024 Feb 21;19(2):e0295589. doi: 10.1371/journal.pone.0295589 (PMC10881014; doi:10.1371/journal.pone.0295589)
Supplement: Attachment — Submitted filename: Comments_to_reviewer_Picciulin_et_al_2023.docx [file pone.0295589.s002.docx]

***Dear Editor***

***please, find below our point-to-point answers to the revision of the manuscript “Rhythmic properties of* Sciaena umbra *calls across space and time in the Mediterranean Sea”, PONE-D-23-21637. All comments have been taken into account, carefully considered and incorporated into the new version of the paper or discussed in the text below. The revisions are listed below, our comments are written in bold italics.***

***Sincerely, Marta Picciulin and co-authors***

Reviewer #1: The authors present interesting and through study of *Sciaena umbra* acoustic signaling behaviour across four different sites in the Mediterranean. The paper is generally well written and follows a clear, linear structure and narrative. I enjoyed reading the manuscript and would like to thank the authors and the editor for giving me the opportunity to comment on this work.

I think that overall the methodology is solid and the results are well presented. The topic of acoustic communication in fish is definitely understudied, especially when taking into the consideration the richness of fish species and their enormous economic value. I also appreciate that the authors choose to explore the rhythmic structure of the signals, this is indeed a very interesting avenue and a potential fitness indicator which started receiving more attention only recently. So, the paper is timely and offers few great suggestions for future work. I do have few comments but think that the authors should be able to address them with relative ease.

Major comments:
1. One major issue that immediately popped out when first reading the paper is the sub setting of the collected data (L159). The authors selected “Twenty 1-minute sequences …. per dataset”. Since the data was collected by passive acoustic monitoring, over several months, I assume that much more acoustic data was collected. I am very well aware that processing and annotating audio data is extremely time and labor consuming, however I think that the authors should give a more detailed justification for their chosen sample size. Especially I would be very interested in seeing an estimation of the overall call time per site, as it can indicate population density and perhaps have an effect on the signalingbehavior.

***This paper comes from the shared willingness of all co-authors to explore rhythm in a well-studied fish soniferous species, in spite of the lack of dedicated funding for this task. A dedicated, funded project was not available. The data used in this study can and should be considered opportunistic; in fact, they were collected at sea by MB and MP in the contexts of different projects and for different purposes than those analysed in this manuscript. Therefore, sampling designs and recording configurations naturally differ between datasets presented in this paper. As now explained in the ms, we considered different datasets, which include:***

1) ***Trieste***

***a series of 11-hour recordings along the summer period - 10 min wav recordings (over the 24 hours) – (Picciulin et al 2012, 2013)***

***a week of continuous monitoring - 1 min wav recordings collected every 5 min (over the 24 h) (unpublished data - 2021)***

2) ***Mallorca and Crete***

***one month of continuous monitoring - 1 min wav recording collected every 11 min (over the 24 h) (Bolgan et al 2022)***

3) ***Venice***

***three single 10-h acoustic campaigns conducted at 40 listening points in two summers- 5 min wav recordings (Picciulin et al 2020, 2021).***

***The shortest duty cycle (i.e., 1/11in Mallorca and Crete) was our limiting factor for analysing the same time window in all datasets. Along these ‘one-minute’ audio files, the authors had to select the cases in which (i) Sciaena umbra sounds were present, (ii) the recorded pattern was the R-pattern, (iii) a recognizable, single sequence characterized by sounds with the same Signal-to-Noise Ratio was present (very often, two or three sequences were present but they overlapped at some point), (iv) the chosen files were not consecutively recorded in order to avoid pseudo-replication.***

***The above-mentioned considerations explain the chosen sample size reported in this manuscript. We hope that the extended explanation provided in the main manuscript helps to clarify the ‘opportunistic nature’ of our study design.***

2. Another related concern would be the uniformity of sampling throughout the data collection periods. Since the authors mention that breeding season (August) is associated with an increase in signaling rhythm, they might want to provide some information on how balanced their sampling was. Looking at Fig4 it seems that most of the August data is coming from the Trieste site. Venice site has half of its data in June, large gap in July and more data in August again. Just from eyeballing the scatter plot, Trieste shows an increase in rhythm but other sites might not. Adding more July samples from Venice might strengthen the regression line for the month effect. I wonder if the authors considered fitting a model with interaction terms and random effects to allow a better fit and a better explanation of the relationships among regressors. Or they could try and balance the dataset by making sure that the 4 sites are more or less equally represented and sampled periodically throughout the period of interest.

***We agree with the referee from a theoretical point of view; however, we refer the reviewer to our previous explanation about the opportunistic nature of our sampling design. This comment has provoked discussion and thinking on our part, and we thank the reviewer for this; as a result, we have now changed the modelling approach. We now use General Additive Models (GAM), which are better suited to our dataset for several reasons: 1) after re-evaluating the qq. plots, not only for the significant parameters in the Ordinary least square regression (OLS), but also the variables excluded from the final model, we came to the conclusion that the data does fit well enough with linear model assumptions. GAMs don’t need the data to adhere to linearity. Furthermore, by using GAMs, we could include both categorical and continuous variables, and as suggested by the reviewers, we could explore also the interactions between variables (i.e., month and day). Relevant diagnostics plots for this approach, as well as a more detailed description of model selection, are now included in the main manuscript, and in the newly added supplementary materials.***

***Furthermore, we extended our discussion to both highlight the limitations of our study (including this point) and call for dedicated studies, investigating the full season. We hope that the ms is now more clear and statistically robust.***

3. Vessel traffic analysis: In L217 the authors mention that 6 different vessel categories were considered but the model output (Table2) only shows trade and fishing vessels. Were the other vessel categories omitted in the model selection process? From Fig5 it seems that the trade and fishing categories were mainly represented on Venice site. This site also had the lowest acoustic richness and highest water depth. So, as authors clearly mention in the discussion (L438) the effect if the vessel noise is very inconclusive and other factor could be driving the higher signalling rhythms in their study species. Reading the Abstract I expected a much stronger support for the anthropogenic effects on the signaling rhythm but after going through the results, I no longer see this as as a major finding of the study. Perhaps the authors would consider toning it further down in the Abstract.

***We do not think that vessel noise role is “very inconclusive” based on the presented analysis; however, following this comment, we have slightly changed the abstract to better emphasised that vessel noise appears as one of the factors affecting the IOI, but that this is not the only factor.***

***In the abstract, in particular, it is stated: “statistically, the beat frequency was found to be dependent on the season (i.e. month of recording) and potentially influenced by the presence of soniferous competitors and human-generated underwater noise”. It seems to us that the vessel noise role is not particularly stressed as compared to the other mentioned factors. In light of this, we still slightly changed the abstract, by eliminating the mention of the underwater noise in the very last sentence. We hope that now the manuscript is more clear.***

I have a perhaps naïve suggestions (since I know very little about marine vessel noise pollution levels), would it make sense to use the tonnage of the vessel as a proxy for noise levels instead of the vessel type? It might provide one continuous variable, instead of 6 categories, potentially improving statistical power. This is off course only relevant if larger vessels actually generate more noise pollution.

***Thanks for the suggestion! Unfortunately, we do not have this information for all the considered vessels categories and, therefore, we can’t use this approach.***

4. L413 here the authors mention that 27% of the rhythm variability are explained by the three factors (month, presence of other fish and vessel density). In L258 the same 27% are attributed to the month of the recording (the breeding season) only. Overall it was not clear to me what procedure the authors used to estimate the contribution of the different variables to the changes in the signalling rhythm. Also, more generally, while I am not a statistician, I think that the paper can benefit from more details on the process of model diagnostics and selection. The authors mention in L222 that the model assumptions were checked by visual inspection. I think that adding a supplement with the data, full model design, diagnostic plots and model selection steps can help the readers to better assess the data and the results.

***Thank you for this comment. We carefully considered the model presentation and choices again and decided, also based on both reviewers’ comments to change the modelling approach. We now use General Additive Models (GAM), which are better suited to our dataset for several reasons: 1) after re-evaluating the qq. plots, not only for the significant parameters in the Ordinary least square regression (OLS) but also the variables excluded from the final model, we came to the conclusion that the data does fit well enough with linear model assumptions. GAMs don’t need the data to adhere to linearity Furthermore, by using GAMs, we could include both categorical and continuous variables, and as suggested by the reviewer, we could explore also the interactions between variables (i.e., month and day). We can now explain 67% of the variability in the data instead of 27% with the former approach. Relevant diagnostics plots for this approach, as well as a more detailed description of model selection, are now included in the main manuscript, and in the newly added supplementary materials. This modelling approach should have been chosen from the beginning and we thank the reviewers for helping us find a more suitable solution.***

Minor comments:

5. L79-80 This sentence might benefit from rephrasing. It is not clear variable in number of what - Rhythmic categories, pulses or variable number of sounds?

***This has been corrected. Thank you.***

6. L102 – here authors state that “nothing is known about how the rhythmic properties of fish sounds change…..” but the previous paragraph actually has a very nice summary of previous work linking rhythmic properties of fish sounds to environmental and biological factors. I would tone down the claim a bit.

***This has been rephrased as well.***

7. L125 – The Trieste data collection: this is not a continuous 10-year monitoring but two sampling efforts in 2009 and 2019-2020. I feel that "a time span of 10 years" is a misleading phrasing in this case

***This has been rephrased.***

8. L128 - Is number of different sound types is indication of different fish species?

***Thanks for pointing out this lack of clarity. This is not always the case, as some fish species can emit more than one sound type in relation to different behavioural and ontogenetic contexts. When it comes to recording collected at sea, some fish sound types can be identified at the species level (and can therefore be attributed to a specific species, allowing to estimate the number of species at site), while others cannot yet be identified at the specific level. Although the species-specificity and the specific identity of these latter sound types cannot (yet) be drawn, several studies indicated that higher fish acoustic richness (i.e. higher number of fish sound types) corresponds to higher specific diversity (see Desidera’ et a;. 2019, for example), regardless if all fish sound types in a specific vocal community have or have not been identified at the specific level. In the case of this study, all fish sound types were identified in some locations (e.g. Trieste and Venice). In others, like for example Mallorca and, especially, Crete (i.e. site with the highest fish acoustic richness in this study), most of the fish acoustic community composition remain unknown. We changed the text to better explain this point.***

9. L199 - the word spacing is off here

***Corrected.***

Reviewer #2: Rhythmic properties of Sciaena umbra calls across space and time in the Mediterranean Sea, by Marta Picciulin, Marta Bolgan and Lara Burchardt,

It is a very interesting manuscript that shows results on the sound production rhythms of an species of Sciaenid.
These results, in my opinion, are original to be published in Plos One, although the authors have a previous publication where they partially describe these results, in this manuscript the authors detail the results in this species in greater detail. It is pioneering work that talks about the rhythms of sound production in a fish.
Although the manuscript presents a very good representation of the data and its statistical analysis, I think it is missing some citations that are important and will give the manuscript a better strength.
My detailed comments are attached.
In short, I think it is a good manuscript to be published, which needs a minor revision.
Good Luck, Javier S. Tellechea.

Introduction
Line 44-46. This sentence is not clear, 30 times compared to what?

***We are sorry for the confusion. 30 times is not a comparison here, but just the number of times this trait was suggested to have evolved independently by the cited studies. We slightly rephrased this to “[…] production has evolved independently over 30 times […]”***

Line 73. Like other Sciaenids (Tavolga, 1964; (Mok and Gilmore, 1983; Fine et al., 2004; Ramcharitar et al., 2006; Tellechea et al., 2010)

***These references have now been added.***

Line 62. Define the R pattern.

***This has been defined***

Line 76. “The acoustic R-pattern occurs more frequently at dusk…” in which species? Not in all species the choirs are at dusk, see Tellechea et al 2011.

***This refers only to S. umbra; it has been now specified in the ms.***

Line 88. See the paper about sound and wáter temperature :
Connaughton, el al 2000, Holt 2002.

***These references have been now added. Thank you for the suggestion.***

Line 90-93. Here it is necessary to cite articles where periods of fish sound production are described. (Mok & Gilmore 1983; Connaughton & Taylor 1995; Locascio & Mann, 2008

***These references have been added, too.***

Methods
Line 144. “Colmar SRL, La Spezia, IT”, It is not clear to me why this location followed by the description of the power...

***This information has been provided for all the manufacturers (ie. Loggerhead Instruments, FL, USA; Tascam, Montebello, CA; Reson, A/S, Salangerup, Denmark)***

Results.
Table 2. The reason for the astrisk in the value 0.018 must be explained in the legend of the table.

***An explanation for the asterisks has now been added to the table reporting the modelling results, thank you for pointing this out. Please note that the modelling approach, and, therefore this table, has changed. We now use General Additive Mixed Models instead of ordinary least square linear regression. Upon careful consideration of all parameters, we decided that too many variables did not meet the prerequisites for linear modelling for the approach to really be feasible. GAMs are more complex than linear relationships, and can deal with both continuous and categorical variables. Please also see comments on the modelling approach above. The new approach is detailed in the newly added Supplements.***

Discusion
Line 310. Add quotes that are importante in fish vocal activity:
(Fine et al 1977; Lobel and Mann, 1995; Lobel 2002; Rountree et al., 2006; Luczkovich et al., 2008;Tellechea et al., 2010; Amorim et al., 2015)

***These references have been now added, thank you again for the helpful suggestion of additional literature.***

Line 365. More quotes must be add: Saucier, and Baltz.(1993; .Ladich and Fine, 2006; Mok et al., 2009; Tellecheaet al., 2010a, Tellechea and Norbis, 2012

***These references have been now added.***

Line 368-372. yes, but there are variations such as in the case of Pogonias cromis (Locacio and Mann 2009) and Pogonias courvina (Tellechea et al 2011 and Tellechea et al 2022, where the reproduction sound is modulated... it would be interesting if they add this exception, since they are the only two species that present this characteristic.

***We now mention these cases as well as the suggested references. Thank you for pointing out this exception!***

Line 378. In addition to (Luczkovich et al. 1999), add Connaughton, M. A., and Taylor, M. H. (1995).and Tellechea, J. S., Bouvier, D., and Norbis, W. (2011).

***These references have been now added, too. Thank you.***
